# Development of Nontoxic Peptides for Lipopolysaccharide Neutralization and Sepsis Treatment

**DOI:** 10.1021/acsptsci.4c00033

**Published:** 2024-05-21

**Authors:** Avner Fink, Daniel Ben Hur, Naiem Ahmad Wani, Hadar Cohen, Li-Av Segev-Zarko, Christopher J. Arnusch, Yechiel Shai

**Affiliations:** † Department of Biomolecular Sciences, Weizmann Institute of Science, Rehovot 76100, Israel; ‡ Department of Desalination and Water Treatment, Zuckerberg Institute for Water Research, Jacob Blaustein Institutes for Desert Research, Ben-Gurion University of the Negev, Sede-Boqer Campus 8499000, Israel; § MilliporeSigma Life Science, Kiryat Hamada 13, 9777613 Jerusalem, Israel

**Keywords:** peptide design, host defense peptides, lipopolysaccharide (LPS) neutralization, sepsis, septic shock, macrophage activation

## Abstract

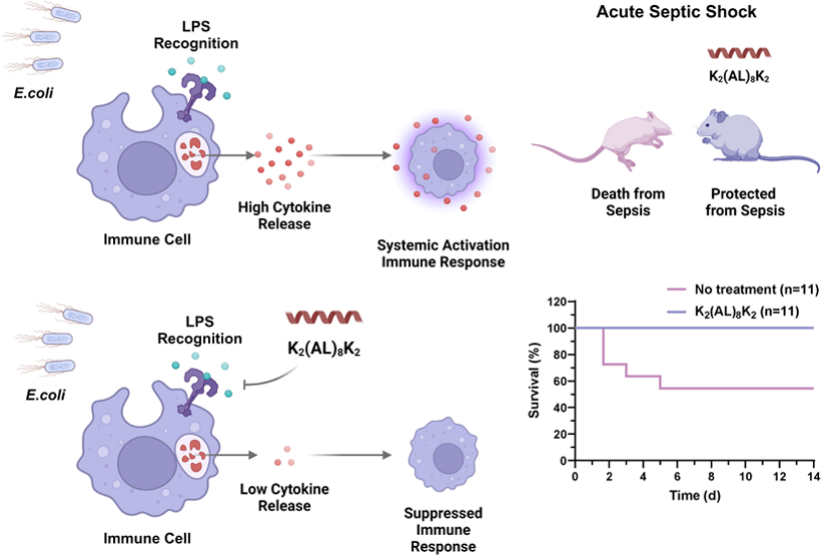

Host defense peptides (HDPs), also named antimicrobial peptides (AMPs), are increasingly being recognized for serving multiple functions in protecting the host from infection and disease. Previous studies have shown that various HDPs can also neutralize lipopolysaccharide (LPS, endotoxin), as well as lipoteichoic acid (LTA), inducing macrophage activation. However, antimicrobial activity is usually accompanied by systemic toxicity which makes it difficult to use HDPs as antiendotoxin agents. Here we report that key parameters can uncouple these two functions yielding nontoxic peptides with potent LPS and LTA neutralization activities in vitro and in animal models. The data reveal that peptide length, the number, and the placement of positive charges are important parameters involved in LPS neutralization. Crucially, the peptide exhibited a separation between its membrane-disrupting and antimicrobial properties, effectively decoupling them from its ability to neutralize LPS. This essential distinction prevented systemic toxicity and led to the peptide’s complete rescue of mice suffering from severe septic shock in two distinct models. Strong binding to LPS, changes in structure, and oligomerization state upon LPS binding were important factors that determined the activity of the peptides. In the face of the increasing threat of septic shock worldwide, it is crucial to grasp how we can neutralize harmful substances like LPS. This knowledge is vital for creating nontoxic treatments for sepsis.

Lipopolysaccharide (LPS) sensing by toll-like receptor 4 (TLR4) is crucial in early responses to infection, where an uncontrolled LPS response gives rise to excessive localized inflammation, such as that found in infected wounds, but also in severe systemic responses to infection.^2,3^ LPS and lipoteichoic acid (LTA) are recognized as pathogen-associated molecular patterns (PAMPs) by pattern recognition receptors such as toll-like receptors (TLRs).^4^ These receptors are expressed on innate immune cells, mainly by mononuclear phagocytes (monocytes and macrophages). Their activation by PAMPs results in the secretion of pro-inflammatory cytokines such as tumor necrosis factor-α (TNF-α), interleukin 6 (IL-6), and IL-1β.^2^ Although this is a normal and beneficial response toward an invading pathogen, an unbalanced or an overstimulation of this system can lead to sepsis, organ failure, and death.^5−7^ Host defense peptides (HDPs), also named antimicrobial peptides (AMPs), are central effector molecules of the innate immune system and are produced by the host as an initial response to combat pathogen infections.^8−10^ Originally, these peptides were characterized for their ability to target and lyse bacterial membranes of both Gram-negative and Gram-positive bacteria.^11−14^ In recent years, there is growing evidence that some HDPs can neutralize the cytotoxicity of LPS. For example, lactoferrin, polymyxin B, temporins, and magainin analog MSI-78 neutralize LPS, while other HDPs such as magainin do not have this ability.^15−19^ On the other hand, other studies showed that de novo-designed peptides that share some of the classical HDP characteristics can neutralize LPS toxicity as well.^20,21^ The mode of action studies for both native and de novo peptides revealed different mechanisms of LPS neutralization depending on the type of AMPs used. Most studies examined the interactions of the peptides with lipid A, the hydrophobic anchor of LPS to the membrane. These studies emphasized the interaction of the positive charges on the peptides with the phosphate head groups on the lipid A as well as the hydrophobic interaction of the peptide backbone with the acyl chains of the lipid A.^15,22,23^ Other research groups have focused on the aggregation state of LPS upon association with neutralizing HDPs.^21,24,25^ We have previously focused on hydrophobicity and charge as more general parameters defining LPS neutralization.^26,27^ Taken together, it has become clear that endotoxin neutralization properties of peptides are much more complex than simple binding to LPS. To date, despite extensive research, the exact properties correlating LPS neutralization and antimicrobial activity of peptides are still not fully understood.^28,29^ Previous studies conducted in our laboratory demonstrated that transmembrane domain (TMD) TLRs inhibit the immune response.^30−33^ These TMD-TLR-derived peptides share a common feature, characterized by a high hydrophobic core and positively charged lysines at the termini of the peptides. Moreover, given alanine’s prevalence in soluble and membrane proteins, mixed alanine-leucine sequences were selected to mimic simplified transmembrane helical domains, assuming favorable interactions with the lipid bilayer’s hydrophobic core and adopting α-helical conformations.^34−39^ Armed with this knowledge, a combination of positive and hydrophobic residues, we designed and investigated a series of HDPs toward LPS neutralization and to prevent sepsis. Here we show that the binding and neutralization activity of a peptide can be uncoupled from its antimicrobial activity. We achieved this by synthesizing a series of peptides that included glycine, alanine, valine, leucine, and lysine. This approach allowed us to systematically manipulate physical characteristics, which include peptide length, sequence, charge, and structure. All the peptides were tested for their antimicrobial activity, toxicity, and LPS neutralization in vitro. Importantly, a single dose of a selected nontoxic peptide with high LPS neutralization ability was able to inhibit septic shock in mice induced by purified LPS or by whole heat-killed *Escherichia coli*. Our hypothesis is that these peptides change the rigidity/fluidity of the membrane and therefore affect the signaling.

## Results

### K_2_(AL)_8_K_2_ and K_2_(AV)_8_K_2_ are Nontoxic and Potent LPS and LTA Neutralizers

**Figure 1:**
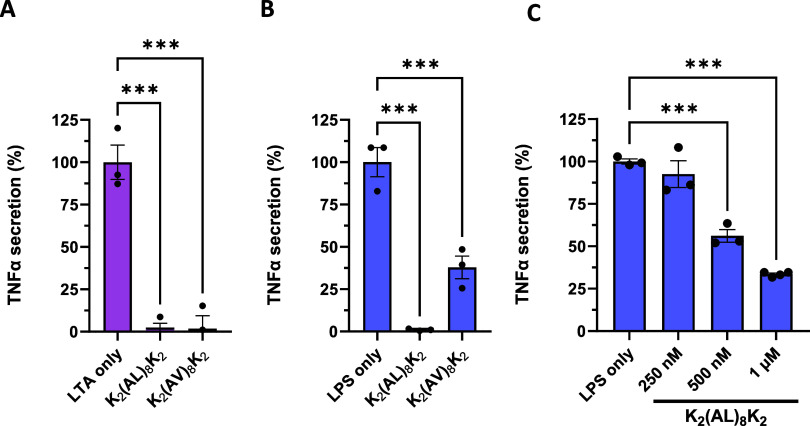
Peptide inhibition of TNFα secretion upon stimulation with (A) LTA and (B) LPS. For both peptides tested, a single concentration of 20 μM was used. (C) Dose-dependent inhibition of TNFα by K_2_(AL)_8_K_2_. The experiments were conducted with either duplicates or triplicates, each with three independent repeats (*n* = 3). Data are presented as means ± standard error of the mean (SEM). Statistical significance was determined using analysis of variance (ANOVA) tests, with significance levels denoted as follows: *p ≤ 0.05, **p ≤ 0.01, and ***p ≤ 0.001.

Initially, we designed hydrophobic positively charged peptides composed of eight alanine/leucine (AL) or alanine/valine (AV) repeats and four lysine residues, two on each terminus as indicated in Table 1, that exhibit identical length and charge. The sole distinction lies in the alteration of relative hydrophobicity and hydrophobic moment. The peptides were tested for their ability to inhibit TLR4 and TLR2 activation by LPS and LTA, respectively (Figure 1A,B). Remarkably, the AL peptide was highly potent in inhibiting TNFα release in macrophages treated with either LPS or LTA. This contrasts with the AV peptide, which showed good inhibition for LTA activation (Figure 1A) but only medium inhibition for LPS activation (Figure 1B). Therefore, the AL peptide was examined further and showed a dose-dependent inhibition of macrophage activation by LPS with 50% inhibition at a concentration of 0.5 μM (Figure 1C). In comparison, less hydrophobic peptides with AA (K_2_(AA)_8_K_2_) and GL (K_2_(GL)_8_K_2_) repeats were completely inactive at concentrations up to 20 μM (Figure S1A,B).

**Table 1:** Peptide Designations and Properties

peptide designation and sequence^a^	length	charge	mol. wt	relative hydrophobicity^b^	hydrophobic moment^c^ (μH)
K_2_(AL)_8_K_2_	20	+5	2003	64	0.199
K_2_(AV)_8_K_2_	20	+5	1890	58	0.18

a All of the peptides are amidated at their C-termini and have the same charge and length.

b Relative hydrophobicity is reflected by the percent of acetonitrile at the retention time.

c Calculated hydrophobic moment (μH) of HDPs.^1^

### Peptide Length, Net Charge, and Charge Distribution Affect the LPS Neutralization

**Figure 2:**
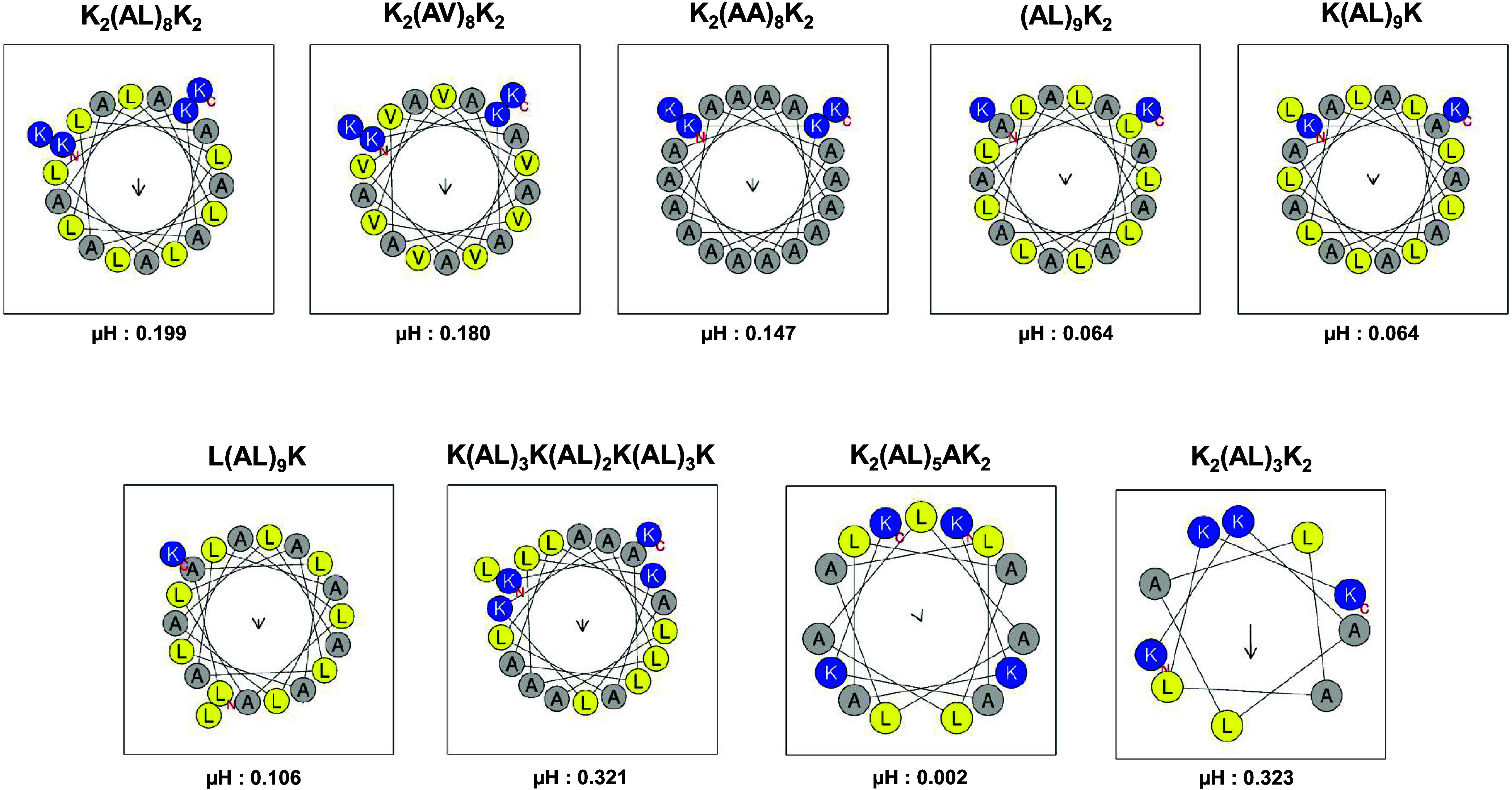
Helical wheel projections of the K_2_(AL)_8_K_2_ and its analogs. Positively charged residues are shown in blue, hydrophobic residues are shown in yellow, and Ala are shown in gray. The arrows indicated the orientation of hydrophobic moment (μH).^1^

**Figure 3:**
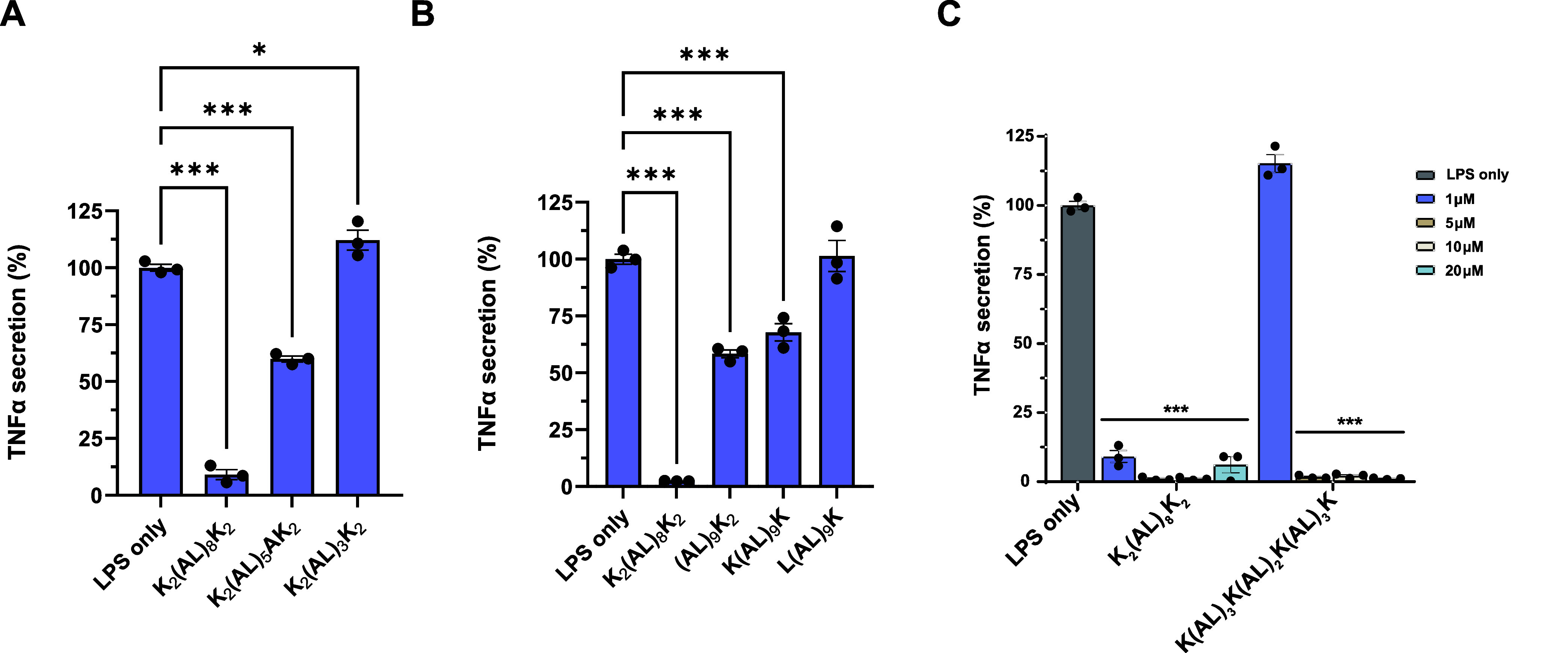
Peptide inhibition of TNFα secretion upon stimulation with LPS. (A) Different lengths of peptides compared with a set charge (+5). (B) Different number of charges compared with a set length (20-mer). For both experiments, peptide concentration used was 20 μM. (C) Charge separation along the peptide compared with a set charge (+5) and length (20-mer). Peptides were added to cells in a dose-dependent manner with concentrations ranging from 1 to 20 μM. The experiments were conducted with either duplicates or triplicates, each with three independent repeats (*n* = 3). Data are presented as means ± SEM. Statistical significance was determined using analysis of variance (ANOVA) tests, with significance levels denoted as follows: *p ≤ 0.05, **p ≤ 0.01, and ***p ≤ 0.001.

The crucial factors related to LPS neutralization activity were subjected to additional testing. Based on the sequence of the most active peptide K_2_(AL)_8_K_2_, we produced a series of peptides and modified their length, net charge, and change position in a systematic manner. Examples include lysines present on both the C and N termini, exclusively on the C-terminus, and in certain peptides, lysines are distributed throughout the peptide (Table 2). This modified series of peptides allowed us to investigate the peptide parameters to gain LPS neutralization activity. Figure 2 shows the helical wheel structure and hydrophobic moment of K_2_(AL)_8_K_2_ and its analogs. All the peptides were evaluated for their toxicity and capacity to neutralize LPS. Surprisingly, although these peptides are hydrophobic and positively charged, they did not show any cytotoxicity or antimicrobial activity, with the exception of K(AL)_3_K(AL)_2_K(AL)_3_K that showed both antimicrobial activity and moderate toxicity (Table 2). This is an indication of the importance of charge distribution along the peptide sequence for antimicrobial activity. The data revealed that the peptides K_2_(AL)_8_K_2_ and K(AL)_3_K(AL)_2_K(AL)_3_K with +5 net charge gave a strong, concentration-dependent inhibition of TNFα (Figure 3A,C), whereas the peptide K_2_(AL)_3_K_2_ was significantly inactive at 20 μM, the highest concentration tested (Figure 3A). Specifically, K_2_(AL)_3_K_2_ treatment resulted in more TNFα secretion than the untreated (Figure 3A). By maintaining the same number of charges (+5) across peptides of different lengths (20-mer, 15-mer, and 10-mer), we demonstrated that the 20-mer peptide is optimal for cytokine inhibition activity (Figure 3A). Keeping the length of the peptides to 20-mers with different charges (+5, +3, and +2) revealed that the peptide with a charge of +5 was the most active in cytokine inhibition, whereas the peptide with +2 charge L(AL)_9_K was inactive (Figure 3B). In addition, we investigated the importance of the peptide hydrophobicity in neutralizing LPS. We replaced Leu with Ala and Ala with Gly to decrease the peptides’ hydrophobicity and to examine their effects on the immune response (Table S1 and Figure S1). Specifically, in peptide K_2_(AA)_8_K_2_ in which Leu was substituted with Ala, was utilized to elucidate Leu’s contribution to the activity (Figure S1A). Lastly, K_2_(GL)_8_K_2_ with Ala and Gly substitutions was used to examine Ala’s role in the inhibition activity (Figure S1B). The results demonstrated that none of the peptides showed inhibition of the cytokines at any of the tested concentrations (Figure S1A,B). K_2_(AL)_8_K_2_ was expected to adopt an α-helix structure. Thus, we investigated whether d-amino acid incorporation could neutralize LPS and tested the effect of a peptide with d-amino acid substitution (Table S1). Incorporating peptides with altered chirality can affect peptide structure and activity, and literature suggests that d-amino acid incorporation can disrupt the α-helix structure.^40^ The diastereomeric peptide with 4 d-leucines was still able to neutralize LPS cytotoxicity to some extent but was less active than the parental K_2_(AL)_8_K_2_ peptide (Figure S2). Note that the exact arrangement of the charges on the termini did not affect this property, as two different peptides with +3 net charge gave the same activity. From these results, we concluded that optimal hydrophobicity must be achieved to get an improved immune response.

**Table 2:** Peptide Designations, Antimicrobial Activity, and Toxicity of Peptides (μM)

peptide designation and sequence^a^	length	net charge	mol. Wt	*E. coli* (ATCC 25922)	*S. aureus* (ATCC 6538P)	LC_50_ macrophages RAW 264.7
K_2_(AL)_8_K_2_	20	+5	2003	>100	>100	>100
K_2_(AV)_8_K_2_	20	+5	1890	>100	>100	90
(AL)_9_K_2_	20	+3	1931	>100	>100	>100
K(AL)_9_K	20	+3	1931	>100	>100	>100
L(AL)_9_K	20	+2	1916	>100	>100	>100
K(AL)_3_K(AL)_2_K(AL)_3_K	20	+5	1988	25	12.5	25
K_2_(AL)_5_AK_2_	15	+5	1522	>100	>100	>100
K_2_(AL)_3_K_2_	10	+5	1088	>100	>100	>100

a All peptides are amidated at their C-termini.

### High LPS Affinity and a High Oligomerization State Observed in Peptides that Neutralize LPS

**Figure 4:**
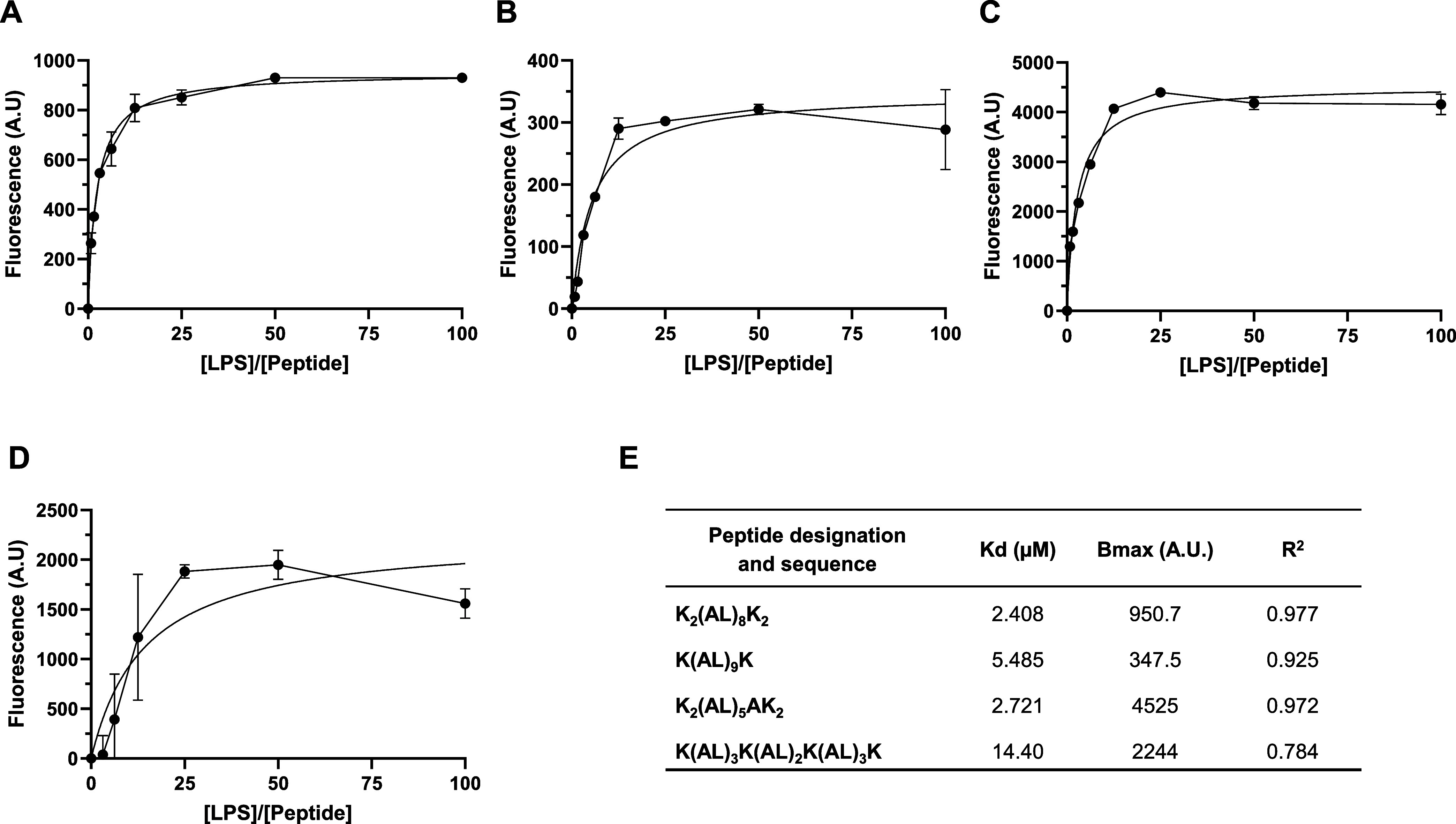
Binding affinity of peptides. Different concentrations of LPS (from 1.56 to 100 μM) were added to NBD-labeled peptides (1 μM), and the fluorescence was recorded. (A) K_2_(AL)_8_K_2_; (B) K(AL)_9_K; (C) K_2_(AL)_5_AK_2_; (D) K(AL)_3_K(AL)_2_K(AL)_3_K; and (E) binding parameters table. NBD excitation was set on 467 nm, and emission was set on 530 nm. Data are presented as means ± SEM. *K*_d_ and binding equilibrium (*B*_max_) were determined using non-linear least squares (NLLSQ) analysis.

With the aim to examine possible mechanisms of action for LPS neutralization, we first measured the affinity of the different peptides toward LPS. To this end, we titrated LPS to a solution of NBD-conjugated peptides. We measured the increase in the fluorescence emission since NBD emission is sensitive to changes in the proximal hydrophobic environment (Figure 4). The most active peptide K_2_(AL)_8_K_2_ affinity for LPS is *K*_d_ = 2.408 μM (Figure 4A). Replacing two lysines, one from each terminus with AL resulted in the elevation of *K*_d_ to 5.485 μM, highlighting the importance of KK at the terminus of the peptide (Figure 4B). The reduction of the hydrophobic core in peptide K_2_(AL)_5_AK_2_ leads to the *K*_d_ = 2.721 μM, resulting in ∼50% inhibition at 20 μM compared to K_2_(AL)_8_K_2_, which showed ∼90% inhibition at the same concentration (Figure 4C). The scrambled peptide K(AL)_3_K(AL)_2_K(AL)_3_K, in which positive charge is equally spread over the hydrophobic core, reduces the peptide LPS binding with the *K*_d_ = 14.4 μM (Figure 4D). A closer examination of the LPS titration curves revealed that the value of *B*_max_ is different for each peptide (Figure 4E). This may reflect the oligomerization state of the peptide when bound to LPS quenching of the fluorescent signal due to a highly oligomerized peptide probably resulting in a lower *B*_max_.

### Secondary Structure of Neutralizing Peptides Improved in LPS Environment

**Figure 5:**
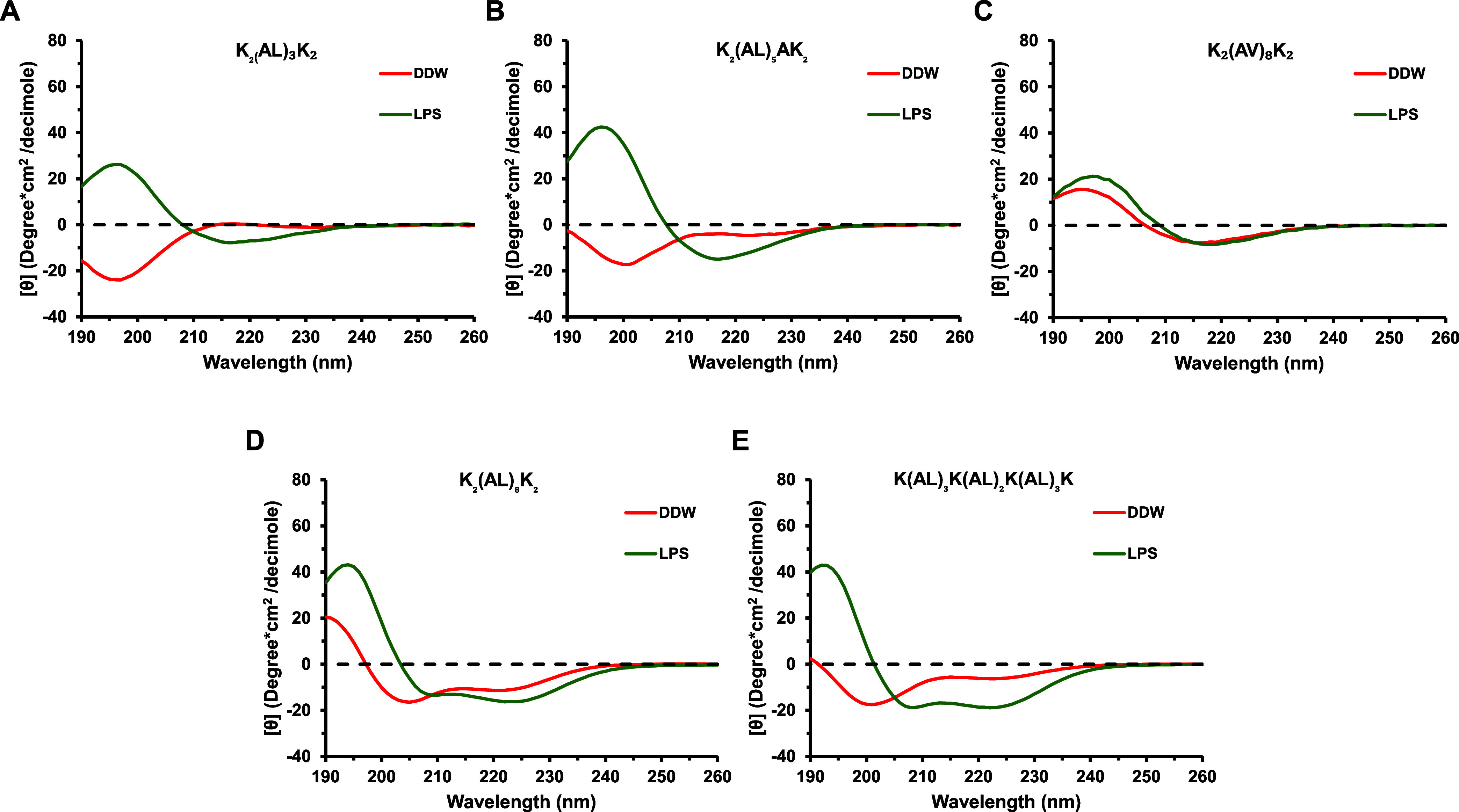
Secondary structures of selected peptides without LPS (red) and with LPS (green). (A) K_2_(AL)_3_K_2_; (B) K_2_(AL)_5_AK_2_; (C) K_2_(AV)_8_K_2_; (D) K_2_(AL)_8_K_2_; and (E) K(AL)_3_K(AL)_2_K(AL)_3_K. Spectra of peptides were scanned at a concentration of 50 μM in double-distilled water (DDW) with or without 50 μM of purified *E. coli* LPS (Sigma-Aldrich) at pH 7.

To test the effect of LPS on the structure of the different peptides, circular dichroism (CD) was performed with and without LPS. LPS improved the structure of the peptides or even induced a structure for some of the peptides. For example, peptides K_2_(AL)_3_K_2_ and K_2_(AL)_5_AK_2_ adopted an antiparallel β-sheet structure. The scrambled peptide K(AL)_3_K(AL)_2_K(AL)_3_K exhibited a random coil structure in solution and adopted an α-helical structure in LPS. In contrast, the parental peptide K_2_(AL)_8_K_2_ adopted a helical structure in solution and LPS (Figure 5). Table 3 depicts the results aligned with the anticipated secondary structure of the peptides, as indicated by the values generated through BESTSEL analysis software.^41^ Interestingly, some peptides adopted α-helix and others β-sheet structures, suggesting that a specific structure is not a prerequisite for LPS neutralization (Figure 5 and Table 3). On the other hand, selected examples suggested that peptides with an α-helical structure are more active than those with a β-sheet structure. Peptides K_2_(AL)_8_K_2_ and K(AL)_3_K(AL)_2_K(AL)_3_K are highly active and adopt an α-helical structure in LPS, while peptide K_2_(AL)_5_AK_2_ showed lower activity and adopt a β-sheet conformation upon interaction with LPS. Additionally, the peptide K_2_(AV)_8_K_2_ showed reduced activity and maintained a β-sheet conformation both before and after the addition of LPS, suggesting no alteration in its secondary structure. Furthermore, CD analysis can also be used to confirm the oligomerization states that were observed in the binding experiments. Measuring the ratio between 222/208 serves as an indication of the oligomerization state of the peptides. Values of ∼0.8 indicate a monomer, and values ∼1 and higher indicate an oligomeric state.^42^ The most active peptide K_2_(AL)_8_K_2_ was calculated to exist predominantly in a monomeric state in pure solution (222/208 = 0.82), whereas an oligomeric state was observed in a solution containing LPS (222/208 = 1.05). K(AL)_3_K(AL)_2_K(AL)_3_K also showed an oligomeric state in the LPS solution (222/208 = 1.00). These results, along with the NBD-binding assays, indicate that LPS-mediated oligomerization can be an essential parameter for the neutralization activity of a specific peptide.

**Table 3:** Predictions for the Secondary Structure of the Peptides by BESTSEL Analysis Software

	K_2_(AL)_3_K_2_		K_2_(AL)_5_AK_2_		K_2_(AV)_8_K_2_		K_2_(AL)_8_K_2_		K(AL)_3_K(AL)_2_K(AL)_3_K	
	DDW	LPS	DDW	LPS	DDW	LPS	DDW	LPS	DDW	LPS
α-helix	0	22	18	21.3	4.7	0	65	64.4	7.9	47.1
antiparallel β-sheet	67	21	73	37.8	71.1	81.6	0	8.5	24.8	3.6
parallel β-sheet	0	57	0	26	24.2	18.4	0	0	0	5.8
β-turn	33	0	8.8	0	0	0	0	7.5	18.2	11.3
random coil	0	0	0	14.9	0	0	35	19.6	49.1	32.1

### Transmission Electron Microscopy

**Figure 6:**
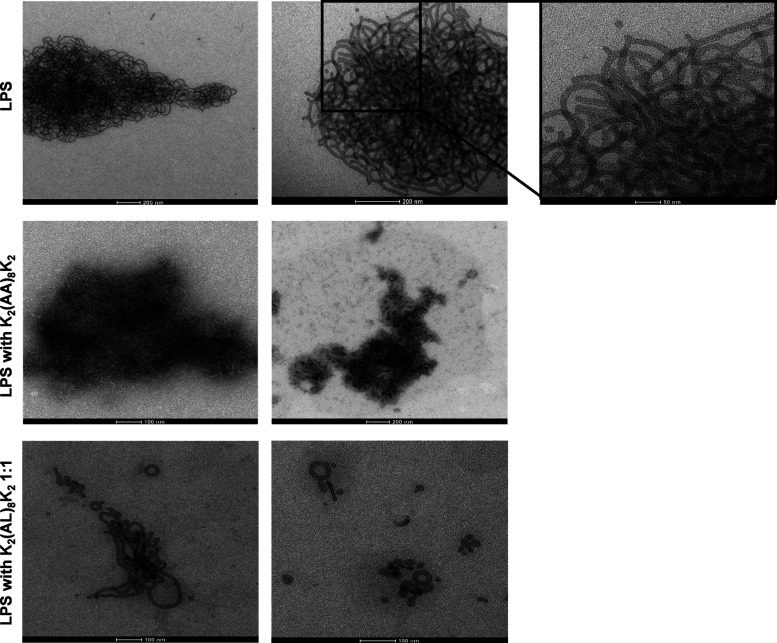
TEM imaging of the LPS in the absence and the presence of the peptides. Scale bar: 100–200 nm.

It is well-known from previous studies that LPS forms aggregates in solution.^27^ We performed a transmission electron microscopy (TEM) analysis to investigate the LPS conformation in the absence and in the presence of the peptides. It was observed that LPS alone, in 10 μM concentration, appears as long filaments bound together and form clusters (Figure 6). As we showed earlier, the peptide K_2_(AA)_8_K_2_ has no neutralization potential and was used as a control (Figure S1A) . In the presence of the peptide K_2_(AA)_8_K _2_ the LPS forms more condensed and rigid aggregates (Figure 6). As previously disscussed the peptide K_2_(AL)_8_K_2_ has the potential to neutralize LPS. Therefore, we visualized LPS in the presence of the K_2_(AL)_8_K_2_ peptide and it was demonstrated that the structure of the LPS became deformed, appearing more open and form a small “donut″-like structure (Figure 6).

### K_2_(AL)_8_K_2_ Inhibits Severe Septic Shock Development in Mice

**Figure 7:**
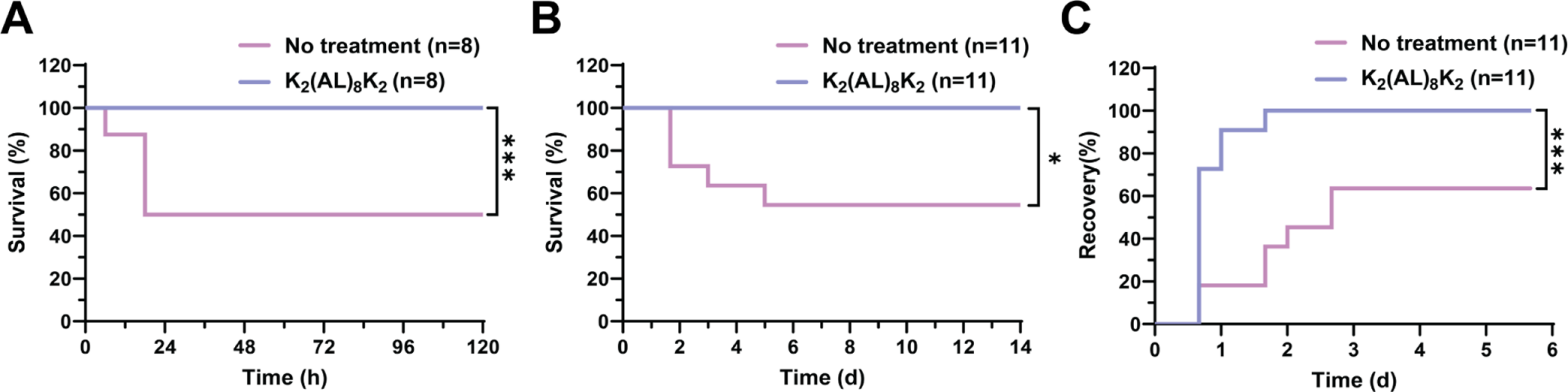
Peptide K_2_(AL)_8_K_2_ protection from sepsis. Sepsis was induced in C57BL/6 mice by i.p. injection of (A) purified LPS (100 ng) with d-galactosamine (40 mg) in 200 μL saline, *n* = 8 or (B) heat-killed *E. coli* (2 × 10^9^ CFU, in 200 μL saline) *n* = 11. Treatment was administered immediately following sepsis induction with 200 μL i.p. injection of K_2_(AL)_8_K_2_ (10 mg/kg). (C) For recovery time from sepsis induced by heat-killed *E. coli*, mice were scored on the severity of sepsis (*n* = 11).

Efficacy testing was performed in two different animal models of septic shock. In the first model, sepsis was induced by injection of purified LPS, whereas in the second model, heat-killed bacteria were used. The peptide K_2_(AL)_8_K_2_ was chosen since it exhibited the most desirable LPS neutralization properties. It inhibited TNFα secretion in stimulated macrophages at a submicromolar concentration, had a strong binding affinity to LPS, and most importantly was nontoxic to cultured macrophages. Initially, this peptide was tested for toxicity in vivo via intraperitoneal (i.p.) administration in C57BL/6 mice (100 mg/kg), the highest dose tested and no lasting adverse effects were observed. At 30 min postinjection, mice appeared and behaved normally. For the first sepsis challenge, C57BL/6 mice were injected i.p. with 100 ng purified LPS (*E. coli* strain 0111:B4) and d-galactosamine (40 mg) and were treated with K_2_(AL)_8_K_2_ peptide. In the untreated group (LPS challenge and saline only), 50% mortality was observed within 48 h (Figure 7A). Surprisingly, a single i.p. injection of the peptide K_2_(AL)_8_K_2_ (10 mg/kg) immediately after the challenge resulted in complete protection of the mice: 100% survival (Figure 7A). In the second model, septic shock was induced with heat-killed *E. coli*. Similar to the first model, only 55% of the untreated mice survived (heat-killed *E. coli* and saline), while the treated mice [heat-killed *E. coli* followed by injection of K_2_(AL)_8_K_2_, (10 mg/kg)] exhibited 100% recovery (Figure 7B). In addition to observing mortality, the recovery of the mice was closely monitored during the first 4 days until no signs of sepsis were observed (Figure 7C). Each mouse was scored twice daily on three physical signs of sepsis: low motility, shivering, and puss secretion from the eyes. The observed recovery time for animals treated with the peptide was significantly shorter than that of the untreated animals.

## Discussion

The increasing interest in developing HDPs as immune modulators has drawn attention in recent years.^43^ Moreover, current antibiotic treatments focus solely on bacteria and do not address the excessive activation of the immune system. This unregulated stimulation can result in an excessive release of inflammatory cytokines, leading to systemic inflammation, blood clotting in blood vessels, and organ dysfunction, as observed in sepsis.^44^ It has been reported that some peptides have been shown to possess additional functions, including anticancer activity, complement activation, direct activation of TLRs, and neutralizing LPS toxicity.^25,27,45−47^ Despite this extensive repertoire of diverse AMPs, there is still limited knowledge on how to separate LPS-neutralizing activity from the systemic toxicity associated with antimicrobial peptides. LPS creates supramolecular aggregates in aqueous environments when exceeding the critical micellar concentration.^48^ These LPS aggregates serve as the biologically active units identified by the host immune system.^49^ Due to its significant role in causing septic shock, researchers are actively seeking new molecules to counteract the exaggerated immune response triggered by LPS. Among these, AMPs, such as MSI-78, LL-37, and amphipathic-d, are appealing due to their ability to interact with and neutralize LPS, offering hope in preventing sepsis.^17,27^ In the present study, we investigated a group of peptides inspired and designed from the TLR-derived peptides and alanine-leucine sequences to assess their capacity to neutralize LPS and evaluate their toxicity. Our findings demonstrated that the effectiveness of LPS neutralization was strongly influenced by peptide hydrophobicity, length, and the number of charges. Specifically, decreasing peptides hydrophobicity (such as substituting leucine with valine or alanine) led to a significant reduction in activity, as showed with peptides K_2_(AL)_8_K_2_ compared to K_2_(AV)_8_K_2_. Moreover, reducing the peptide length led to a decrease in LPS-neutralizing activity, as demonstrated by comparing peptides K_2_(AL)_8_K_2_, the 20-mer, to K_2_(AL)_5_AK_2_, the 15-mer, and K_2_(AL)_3_K_2_, the 10-mer. Similarly, reducing the number of charges led to a significant decrease in efficacy, as demonstrated by the TNFα inhibition activity test comparing peptides K_2_(AL)_8_K_2_ with (AL)_9_AK_2_, K(AL)_9_K, and L(AL)_9_K, wherein K_2_(AL)_8_K_2_ remained the most active peptide. The reduction in positive charges corresponded with a decrease in activity, indicating the importance of charge density in peptide efficacy. Importantly, the distribution of charges influences peptide activity. It has been observed that maintaining a hydrophobic core with positive charge termini is critical for peptide functionality, as demonstrated by K(AL)_3_K(AL)_2_K(AL)_3_K. Furthermore, five representative peptides were chosen to investigate the mode of action. K_2_(AL)_3_K_2_ and K_2_(AL)_5_AK_2_ peptides were selected to represent different sequence lengths, comprising 10 and 15-mer peptides, respectively. The peptide K(AL)_3_K(AL)_2_K(AL)_3_K was chosen due to its composition of the same amino acids but with a distributed charge. Additionally, K_2_(AV)_8_K_2_ was utilized as a less hydrophobic peptide compared to K_2_(AL)_8_K_2_, while K_2_(AA)_8_K_2_ served as an inactive peptide. The investigation into the peptides’ affinity to LPS revealed that decreasing the length of the peptide’s hydrophobic core composed of AL amino acids resulted in a reduction in affinity. Furthermore, the presence of a distributed charge within the peptide led to a decrease in the peptide’s affinity to LPS. The peptide’s affinity to LPS showed differing *B*_max_ values for each peptide. Peptides can exist in various states of aggregation, including monomeric, oligomeric, or even forming larger aggregates like fibrils or amyloid structures. The aggregation state of LPS can impact the binding affinity and mechanism of action either by exhibiting stronger interaction with LPS or might form structures that physically block LPS-binding sites. Quenching of the fluorescent signal, possibly caused by highly oligomerized peptide species, likely contributed to lower *B*_max_ values, indicating variations in the peptide’s oligomerization state upon binding to LPS. The investigation of peptide structures aimed to elucidate the connection between folding form and activity. In solution, K_2_(AL)_3_K_2_ and K_2_(AL)_5_AK_2_ peptides primarily adopt an antiparallel β-sheet, transitioning to approximately 30% α-helix, along with ∼30% antiparallel β-sheet and ∼30% parallel β-sheet upon LPS interaction. Conversely, K_2_(AV)_8_K_2_ exists as an antiparallel β-sheet both in solution and upon LPS interaction. Notably, the K(AL)_3_K(AL)_2_K(AL)_3_K peptide shifts to an α-helix structure upon LPS induction, potentially explaining its high activity, akin to K_2_(AL)_8_K_2_ up to 5 μM. Interestingly, K_2_(AL)_8_K_2_ maintains an α-helix structure regardless of LPS interaction. This structural analysis emphasizes the critical role of the α-helix conformation in the LPS neutralization activity of the AL peptides. Additionally, it indicates that LPS-mediated oligomerization could be a fundamental parameter for this neutralization activity. Following affinity, structural, and oligomerization state experiments, we demonstrated that the peptide’s effectiveness is not solely determined by a single factor. Instead, it results from a complex interaction among these various factors.

In summary, the optimal characteristics for an effective neutralizing peptide involve a high affinity for LPS, α-helical structure, and a robust capacity for oligomerization (Table 4). Because of the dynamic interplay of these parameters, prediction algorithms for optimal peptide sequences would be very complex. However, this study has revealed how key modifications can affect activity and toxicity and this information can serve as a guideline for future design strategies. However, this suggests that LPS binding was not the sole determinant of detoxification according to our LPS-binding assays. We hypothesize that these peptides are involved in an additional mode of action, forming a mesh or network-like structure on the membrane surface, primarily due to their hydrophobic properties. This can be allowed by the interactions between the lysine residues and the negatively charged head groups of phospholipids, which could potentially alter the membrane’s mobility. Moreover, the similarity in the sequence of multidomain peptides (MDPs) may provide clues to an additional mode of action for the AL peptides.^50,51^

**Table 4:** Summary of Important In Vitro Peptide Parameters Measured in This Study

peptide designation and sequence	length	charge	antimicrobial	LPS binding	TNF-α inhibition	toxicity to macrophages	α-helix
K_2_(AL)_8_K_2_	20	+5	no	+++	+++	no	+++
(AL)_9_K_2_	20	+3	no	+	+	no	+
K(AL)_9_K	20	+3	no	+	+	no	±
L(AL)_9_K	20	+2	no	–	–	no	*n*
K(AL)_3_K(AL)_2_K(AL)_3_K	20	+5	yes	++	+	yes	+++
K_2_(AL)_5_AK_2_	15	+5	no	+++	+	no	++
K_2_(AL)_3_K_2_	10	+5	no	–	–	no	++

In recent years, many attempts have been made to reduce sepsis mortality. The mortality rates observed in these models represent well the 30–50% mortality rate observed in hospitals with patients receiving the best supportive care.^5^ Hence, nontoxic compounds that can reduce the pro-inflammatory activity of TLR4 in vivo are of great interest. To this end, we tested the most potent peptide, K_2_(AL)_8_K_2_, in vivo using two different murine models of sepsis. Purified LPS, in combination with d-galactosamine, temporarily suppressed liver function and caused death in ∼50% of the animals within 24 h. The second model involving an injection of only heat-killed bacteria caused death up to 4 days postinjection. The two models showed that the peptide can protect against simple as well as complex challenges. Although the animals died faster with an injection of LPS alone, we believe injecting heat-killed bacteria represents a more realistic challenge. In both cases, we saw that the mice treated with the peptide completely recovered and the active peptide was effective in a more complex challenge involving whole bacteria. We observed very low toxicity in vivo, mice injected with up to 100 mg/kg of peptide, a magnitude of 10-fold higher than the treatment dose, showed no adverse signs of toxicity. This is unique compared to the majority of antimicrobial peptides and de novo designed peptides that exhibit a narrow therapeutic window, where the toxic dose is close to the effective dose, imposing severe limitations for their usage in vivo models.^52,53^ It is crucial to highlight that some peptides exhibit high activity in murine sepsis models and can be highly cytotoxic at low in vitro concentrations.^21^ In contrast, our segregated peptide K_2_(AL)_8_K_2_, demonstrated minimal in vitro cytotoxicity coupled with low in vivo toxicity, suggesting that this particular peptide could offer a more comprehensive therapeutic range.

**Figure 8:**
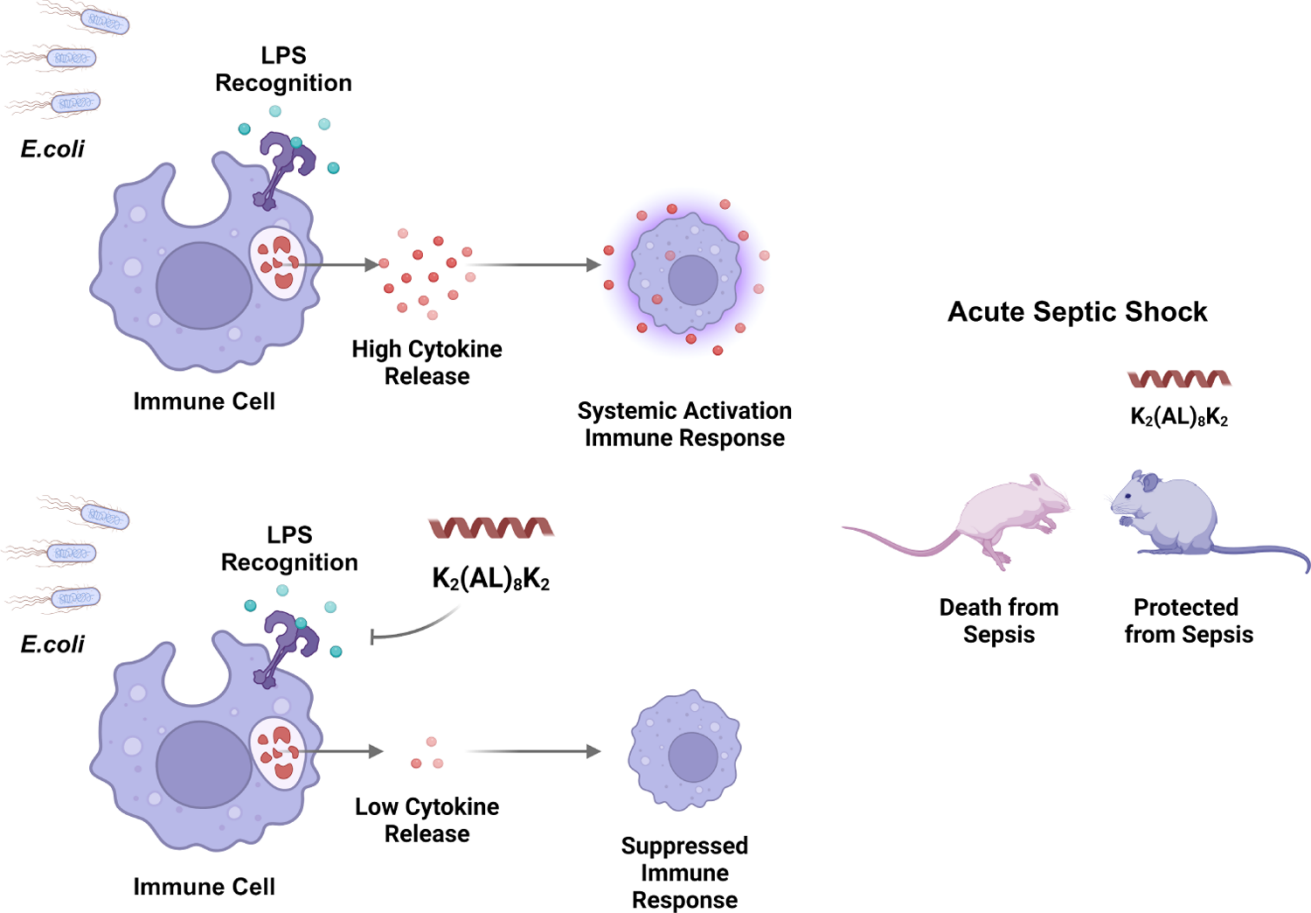
Illustration demonstrating the LPS neutralization mechanism of the K_2_(AL)_8_K_2_ peptide. The peptide neutralizes LPS and blocks LPS recognition by TLR4, leading to inhibition of cytokine release and conferring protection against sepsis. Illustration was created using the Biorender.com platform.

Our findings demonstrated that the K_2_(AL)_8_K_2_ peptide effectively binds to and neutralizes LPS, preventing its recognition by TLR4. This crucial interaction significantly reduces the release of pro-inflammatory cytokines, such as TNF-α, resulting in a suppressed immune response. This inhibition provides robust protection against the overstimulation induced by LPS derived from *E. coli*, ultimately rescuing mice from severe septic shock (Figure 8).

## Conclusions

To summarize, our research introduces a new family of peptides with potent LPS-neutralizing properties, demonstrating low toxicity both in vitro and in vivo. The study identifies the key components necessary for effectively neutralizing LPS and successfully demonstrates that the designed peptide protects mice from acute sepsis in two distinct models. Given the escalating concern of sepsis as a major health issue, these nontoxic peptides hold great promise as potential safe treatments for this challenging condition.

## Materials and Methods

### Peptide Synthesis and Purification

Peptides were synthesized by a 9-fluorenylmethoxylcarbonyl (Fmoc) solid-phase method on Rink amide MBHA resin (Calbiochem-novabiochem, San Diego, California) by using an ABI 433A automatic peptide synthesizer (Applied Biosystems, Foster City, CA) or Liberty Blue Automated MW Peptide Synthesizer 240v (I.S.I., Israel Scientific Instruments Ltd.). Peptides were fluorescent labeled using 4-Fluoro-7-nitrobenzofurazan (NBD, BioChemika). Resin-bound peptides were treated with NBD dissolved in dimethylformamide (DMF), leading to the formation of resin-bound N-terminal fluorophore peptides. 2% *N*,*N*-diisopropylethylamine (DIPEA) was added to the solution and incubated for 1 h. Following the incubation, the resin was washed thoroughly with DMF and then with dichloromethane (CH_2_Cl_2_), dried under nitrogen flow. Peptide synthesis was followed by peptide cleavage from the resin by incubation for 2 h with 95% trifluoroacetic acid (TFA), 2.5% H_2_O, and 2.5% triethylsilane (TES). The crude peptides were washed from the resin using TFA, precipitated using cold diethyl ether, and air-dried. Purification of the crude peptide was performed by reverse phase high performance liquid chromatography (RP-HPLC) (>98%) on a Vydac C4 column (Grace Discovery Sciences, Deerfield, Il). The peptides were characterized by electrospray mass spectroscopy.

### Antimicrobial Assays

The antibacterial activity of the peptides was examined in sterile 96-well plates (Nunc 96-well microtiter plates) in a final volume of 100 μL, as follows. Aliquots (50 μL) of a suspension containing bacteria at a concentration of 10^6^ colony-forming units/mL in culture medium (LB medium) were added to 50 μL of peptide serially diluted in culture medium (100–0.78 μM). Inhibition of growth was determined by the eye after an incubation of 18–20 h at 37 °C. Antibacterial activities were expressed as the minimal inhibitory concentration (MIC), the concentration at which 100% inhibition of growth was observed after 18–20 h of incubation. In these experiments, we have tested the effect of the peptides on *E. coli* (ATCC 25922) and *Staphylococcus aureus* (ATCC 6538P) as representatives of both Gram-negative and Gram-positive bacteria, respectively.

### Cell Culture

All in vitro assays were performed on RAW264.7 murine macrophages (ATCC TIB-71). Cells were grown in Dulbecco’s modified eagle medium (DMEM) supplemented with 10% fetal bovine serum (FBS), l-glutamine, sodium pyruvate, nonessential amino acids, and antibiotics (Biological Industries, Beit Haemek, Israel). The incubator was set on 37 °C with a humidified atmosphere containing 5% CO_2_.

### XTT Cytotoxicity Assays

One ×10^4^ cells per well were grown overnight on a 96-well plate at 37 °C with a humidified atmosphere containing 5% CO_2_. The following day, the media were replaced with 90 μL fresh culture medium and 10 μL solution buffer containing different concentrations of the different peptides. Peptides were serially diluted in culture medium to concentration ranging from 100 to 0.78 μM. The cells were then incubated for 2 h before adding to each well 50 μL of 2,3-bis-2*H*-tetrazolium-5-carboxanilide inner salt (XTT) reaction solution (Biological Industries). Cell viability was determined as described previously.^52,54^ The LC_50_ (the concentration at which 50% of the cells die) for each peptide was obtained from the dose-dependent cell viability curves.

### TNFα Secretion by RAW264.7 in Response to TLR Activation

Two ×10^5^ cells per well were cultured overnight in a 96-wells plate at 37 °C with a humidified atmosphere containing 5% CO_2_. The following day, the media were replaced by fresh DMEM, including all supplements. Peptides were dissolved in dimethylsulfoxide (DMSO) and added to the cells in different concentrations. The final concentration of DMSO was 1% for all groups. Cells were incubated with the peptides for 2 h, and then LPS (TLR4 activator) or LTA (TLR2 activator) was added to the cells at 10 or 500 ng/mL, respectively. The cells were further incubated for 5 h at 37 °C, after which samples of the media from each treatment were collected and stored at −20 °C. TNFα concentration in each sample was evaluated using a mouse TNFα enzyme-linked immunosorbent assay kit (Biosource ELISA, Invitrogen), according to the manufacturer’s protocol. All experiments were done in triplicates.

### LPS-Binding Assays

NBD-labeled peptides (50 μL, 1 μM, PBS (−/−), and 2% DMSO) were added to different concentrations of LPS (50 μL PBS (−/−)) in an opaque black 96-well plate. After 10 min of incubation at room temperature, the fluorescence was measured using an excitation of 467 nm and emission of 530 nm. The data were plotted and *K*_d_ and binding equilibrium (*B*_max_) values obtained using NLLSQ analysis. The NLLSQ fitting was done using the following equation , *K*_d_ was extrapolated from the *K*_a_ values according to the following equation .

### Circular Dichroism (CD) Spectroscopy

CD measurements were performed on an Aviv 202 spectropolarimeter (Applied Photophysics spectropolarimeter, United Kingdom). The spectra were scanned using a thermostatic quartz cuvette with a path length of 1 mm and analyzed for structure proportions using BESTEL software. All measurements were done at 25 °C. The average time recording of each spectrum was 20 s in 1 nm steps in the wavelength range of 190–260 nm. The peptides were scanned at a concentration of 50 μM in DDW with or without 50 μM of purified *E. coli* LPS (Sigma-Aldrich). The average *M*_w_ of LPS used for calculations is 4 kDa.

### Transmission Electron Microscopy

A mixture of 1 μM peptide and LPS at the same molar ratio was incubated for 30 min and deposited on a 400 mesh copper grid coated with a carbon-stabilized Formvar film. After 1 min, excess fluid was eliminated, and the samples were negatively stained with 2% uranyl acetate dissolved in water. After 1 min, the excess uranyl acetate was removed from the grid. The grids were examined using a JEOL JEM 100B electron microscope (Japan Electron Optics Laboratory Co., Tokyo, Japan).

### Ethics Statement

Animal studies were carried out in strict accordance with the Israeli law and the National Research Council guidelines (Guide for the Care and Use of Laboratory Animals 2010). All animal experiments were conducted at the Weizmann Institute of Science and approved by the Weizmann Institutional Animal Care and Use Committee (IACUC permit no. 01190107-4).

### In Vivo Studies

Toxicity was tested by i.p. injection of K_2_(AL)_8_K_2_ (100 mg/kg in 400 μL saline) in female C57BL/6 mice (*n* = 2). Mice were continuously observed for 1 h immediately following injection and once per day for 7 days. To examine the effect of our peptide on acute septic shock driven by LPS hyper-activation of TLR4, we have used murine models as described before.^55,56^ Briefly, 12 weeks old C57BL/6 female mice were treated with 100 ng of LPS injected i.p. in a saline solution (200 μL, pH 6.5) containing 200 mg/mL of d-galactosamine (Calbiochem). Treated mice received one i.p. injection of 10 mg/kg peptide dissolved in saline (200 μL) followed by an injection of LPS. In the second model, heat-killed bacteria were used to induce a lethal septic shock. *E. coli* cells were grown to a mid-log phase (optical density = 0.5) at 37 °C, cooled on ice, and centrifuged at 1200*g* for 10 min at 4 °C. The cells were resuspended in saline for a concentration of 2 × 10^9^ cells in 200 μL and heated at 95 °C for 30 min. Each animal received an i.p. injection of 2 × 10^9^ cells in 200 μL saline followed by an injection of 10 mg/kg peptide dissolved in saline (200 μL). Animals were monitored for survival and signs of sepsis for the next 14 days after LPS injection. For LPS-driven septic shock, *n* = 8. For heat-killed bacteria, *n* = 11. Experiments were done according to the regulations of the animal care facility at the Weizmann Institute of Science.

### Statistical Analysis

Statistical significance was determined using ANOVA tests (*p ≤ 0.05, **p ≤ 0.01, and ***p ≤ 0.001) as implemented in GraphPad Prism 10. In vivo statistical analyses were conducted using Kaplan–Meier survival analysis with GraphPad Prism 10 (*p ≤ 0.05, **p ≤ 0.01, and ***p ≤ 0.001). The results are shown as means ± SEM unless indicated otherwise. Experiments were repeated three times (biological repeats) in triplicate or duplicate.

## Supporting Information Available

Peptide designation and properties, peptide inhibition of TNFα secretion upon stimulation with LPS, peptide inhibition of TNFα secretion upon stimulation of RAW264.7 macrophages with LPS, and parameter definitions for Table 4 are provided in Supporting Information (PDF)
