# Supplementary material for: Development of Nontoxic Peptides for Lipopolysaccharide Neutralization and Sepsis Treatment
Source: ACS Pharmacol Transl Sci. 2024 May 21;7(6):1795–806. doi: 10.1021/acsptsci.4c00033 (PMC11184611; doi:10.1021/acsptsci.4c00033)
Supplement: Supplementary file 1 — pt4c00033_si_001.pdf [file pt4c00033_si_001.pdf]

Supplementary Information

Development of Non-Toxic Peptides for Lipopolysaccharide Neutralization and Sepsis Treatment

Avner Fink[1,3,†], Daniel Ben Hur[1,†], Naiem Ahmad Wani[1,†], Hadar Cohen[1], Li-Av Segev-Zarko[1], Christopher J. Arnusch[2,*] and Yechiel Shai[1,*]

[†] These authors contributed equally to this work

[1] Department of Biomolecular Sciences, Weizmann Institute of Science, Rehovot 76100, Israel.

[2]Department of Desalination and Water Treatment, Zuckerberg Institute for Water Research, Jacob Blaustein Institutes for Desert Research, Ben-Gurion University of the Negev, Sede-Boqer Campus 8499000, Israel.

[3]MilliporeSigma life science, Kiryat Hamada 13, 9777613 Jerusalem, Israel.

*To whom correspondence should be addressed: Yechiel.Shai@weizmann.ac.il and arnusch@bgu.ac.il

Table S1: Peptide designation and properties

peptide designation and sequence [a]	length	charge	mol. wt	calculated hydrophobicity [b]	hydrophobic moment[b] (μH)
$K_2(AA)_8K_2$	20	+5	1665	0.05	0.14
$K_2(GL)_8K_2$	20	+5	1819	0.48	0.19
D,L-$K_2(AL)_8K_2$	20	+5	2003	0.64	0.15
D,L-$(AL)_9K_2$	20	+3	1931	0.80	0.06

[a] All of the peptides are amidated at their C-termini and have the same length. [b] Calculated hydrophobicity and hydrophobic moment (μH) of AMPs were calculated using HeliQuest (http://heliquest.ipmc.cnrs.fr).

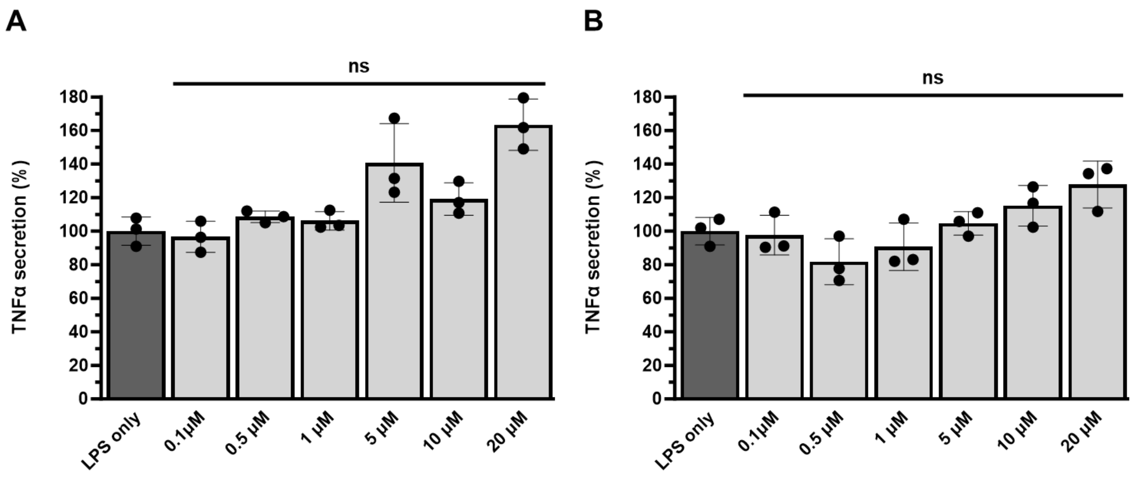

Figure S1. Peptide inhibition of TNFα secretion upon stimulation with LPS. (A) $K_2(AA)_8K_2$. (B) $K_2(GL)_8K_2$. The experiments were conducted with either duplicates or triplicates, each with three independent repeats ($n = 3$). Data are presented as means ± standard error of the mean (SEM). Statistical significance was determined using analysis of variance (ANOVA) tests, with significance levels denoted as follows: $*p \leq 0.05$, $**p \leq 0.01$, and $***p \leq 0.001$.

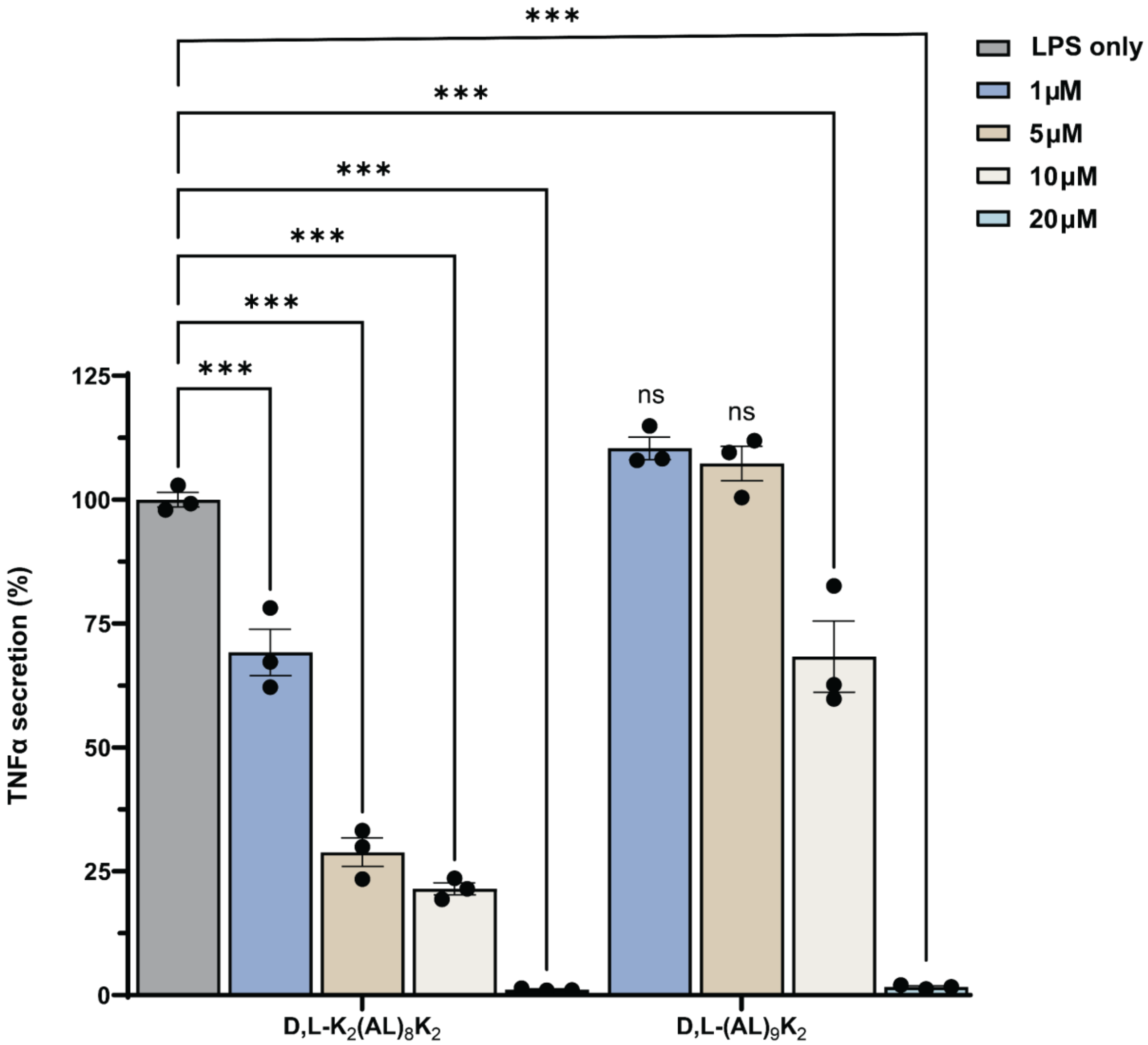

Figure S2. Peptide inhibition of TNFα secretion upon stimulation of RAW264.7 macrophages with LPS. Peptides were added to cells in a dose dependent manner with concentrations ranging from 1 to 20 µM. The experiments were conducted with either duplicates or triplicates, each with three independent repeats (n = 3). Data are presented as means ± SEM. Statistical significance was determined using analysis of variance (ANOVA) tests, with significance levels denoted as follows: *$p \leq 0.05$, **$p \leq 0.01$, and ***$p \leq 0.001$.

Table S2: Parameter definitions for Table 4.

	LPS binding (K_d, μM)	TNF inhibition (%)	α-helix (θ)
+++	1-5	0-20	20-25000
++	5-10	20-40	15-20000
+	10-15	40-60	10-15000
+/-	15-20	60-80	0-10000
-	>20	80-100	-
